# Healthy Ageing and Gut Microbiota: A Study on Longevity in Adults

**DOI:** 10.3390/microorganisms13071657

**Published:** 2025-07-14

**Authors:** Lihua Deng, Jun Xu, Qian Xue, Yanan Wei, Jingtong Wang

**Affiliations:** 1 Department of Geriatrics, Peking University People’s Hospital, Beijing 100044, China; denglihua@pkuph.edu.cn (L.D.); xueqian0719@163.com (Q.X.); heye.wei@163.com (Y.W.); 2 Department of Gastroenterology, Peking University People’s Hospital, Beijing 100044, China; xujun@hsc.pku.edu.cn; 3 Clinical Center of Immune-Mediated Digestive Diseases, Peking University People’s Hospital, Beijing 100044, China

**Keywords:** gut microbiota, frailty, intrinsic capacity, healthy ageing, *Akkermansia muciniphila*

## Abstract

Many studies have focused on ageing and gut microbiota, but the correlation between gut microbiota and physical function in older adults, especially those with longevity, remains obscure and deserves further exploration. In this study we investigated changes in the gut microbiota and the association between gut microbiota and physical function in adults with longevity. This is a prospective observational study. Fifty-one older adults aged ≥ 60 years (including 27 participants aged 90 years and above) were enrolled. Information on clinical data, physical function including intrinsic capacity by Integrated Care for Older People (ICOPE) tool, and dietary habits of participants was collected and analysed. Gut microbiota structure and functional pathways were analysed by Metagenomics. Intrinsic capacity (measured as ICOPE scores) of adults’ longevity (aged 90–98, longe group) was significantly lower than older adults aged 60–89 years (CON group) (5.44 ± 2.15 vs. 6.71 ± 1.46, *p* = 0.017). Gut microbiota of the longe group is enriched in *Akkermansia* and *Bifidobacterium*, which may be beneficial to health. Gut microbiota was closely related to daily milk (including plain milk, flavoured milk with a content of cow’s milk or reconstituted milk of ≥80%, or reconstituted milk or fermented milk with a content of cow’s milk or milk powder of ≥80%) consumption, anxiety, and physical function including grip strength by the Short Physical Performance Battery (SPPB). *Bacteroides plebeius* and *Bacteroides eggerthii* were increased in long-living adults with better physical function. *Escherichia coli* was more abundant in frail young-old adults. Grip strength is positively correlated with the abundance of *Roseburia hominis*, *Eubacterium rectale*, *Eubacterium eligens*, and *Roseburia intestinalis* (*p* < 0.05). Pathways related to amino acid synthesis that include L-isoleucine, L-valine, and L-threonine were over-presented in long-living adults of better physical function. Adults with longevity showed comparable gut microbiota abundance to younger elderly individuals. The gut microbiota of long-living adults showed higher abundance of potentially beneficial bacteria, and the altered bacteria are closely associated with physical function. Changes in the gut microbiota may precede clinical indicators during the process of ageing. Gut microbiota may be a potential biomarker for longevity and healthy ageing. Nutrition and emotional state can be important influencing factors.

## 1. Background

The ageing global population is an important medical, social, and demographic problem [1]. Maintaining physical function and enabling well-being in older age has been defined as “healthy ageing” by the WHO [2,3]. Healthy ageing primarily involves preserving physical function and mental health free from anxiety and expression [2]. Research on healthy ageing has focused on longevity, physical function, and emotional well-being. Gut microbiota may play an important role. Numerous studies have shown that the gut microbiota reflects the influence of the environment on the body, regulates health status and disease risk, plays a regulatory role in the ageing process, and varies with age [4,5,6,7]. Therefore, the gut microbiota is regarded as a “metabolic organ” [8,9]. Pan Shifu et al. [10] found that centenarians’ gut microbiome exhibits youth-associated features in the gut microbiome, with high butyrate-producing bacteria abundance and lower *Enterobacteriaceae* and *Proteobacteria* levels. This suggests gut microbiota and metabolites correlate with longevity. Although many studies have been conducted on ageing and gut microbiota, the correlation between gut microbiota and physical function in older adults, especially those with longevity, remains obscure and deserves further exploration.

Older adults are prone to various chronic diseases that affect their physical function and quality of life. A series of methods include Fried phenotype for frailty, the Integrated Care for Older People (ICOPE) tool for intrinsic capacity, and the Short Physical Performance Battery (SPPB) for physical function. Frailty is a complex age-related condition characterised by a decline in physiological capacity of several organ systems. Adults with frailty are more susceptibility to diseases [11]. Intrinsic capacity was introduced by the World Health Organisation (WHO) [3] and combines all the physical and mental capacities an individual can reveal. Integrated Care for Older People (ICOPE) is a guideline for community-level interventions to manage declines in intrinsic capacity, including cognition, locomotion, vitality, sensory abilities, and psychosocial abilities [12]. The ICOPE tool can be used to quantitatively evaluate intrinsic capacity. The SPPB is a common tool for assessing physical function and is now widely used. Few studies have assessed older adults’ overall health and physical function by comprehensive assessment and examined their correlation with gut microbiota and the role of gut microbiota in healthy ageing.

This study investigated the role of the gut microbiota in healthy ageing by exploring the association between the gut microbiota and frailty, intrinsic capacity, and altered pathways. Information on chronic diseases, clinical indicators, and diet was collected and analysed.

## 2. Materials and Methods

### 2.1. Study Participants

A prospective study was conducted between February 2021 and February 2022, during which long-lived adults (longe group, age range 90 years and above) and young-old adults (control group, age range 60–89 years) were continuously recruited at the geriatric department of Peking University People’s Hospital. Participants visited the geriatric department for regular examinations for chronic diseases. Frailty was defined by the Fried phenotype (FP 2001) [13]. Intrinsic capacity was screened using the ICOPE tool (Table 1) [14]. The Mini-Mental State Examination (MMSE) [15], Short Physical Performance Battery (SPPB) test [16], Mini-Nutritional Assessment Short Form (MNA-SF) [17], Brief 7-Item Self-Report Questionnaire for Generalised Anxiety Disorder (GAD-7) [18], and Patient Health Questionnaire-9 (PHQ-9) [19] were used to assess cognition, locomotion, nutrition, and psychosocial conditions, respectively. MMSE scores less than 27 were defined as mild cognitive impairment (MCI). We calculated a summary intrinsic capacity score by adding responses to all eight dichotomous questions from the five domains (possible range: 0–8). The higher the score, the better the intrinsic capacity. One or more abnormal results on the ICOPE screening tool were defined as impaired intrinsic ability (IC). The study was approved by the ethics committee of Peking University People’s Hospital, and all participants provided written informed consent.

Individuals who met one or more of the following criteria were excluded: inability to move and stand independently from a chair; chronic cardiopulmonary insufficiency preventing normal daily activities (either New York Heart Association Functional Classification III/IV or inability to tolerate a 6 min walk test); renal insufficiency (creatinine clearance < 60 mL/(min·1.73 m^2^)); or the presence of an active malignant gastroenteric neoplastic disease. The exclusion criteria were based on factors known or likely to impact the gut microbiota or those that confound the diagnosis of frailty.

All participants were interviewed to collect data concerning sex, age, education (years), smoking, history of hypertension (HTN) (sitting blood pressure ≥ 140/90 mmHg for two or more times), DM (confirmed by oral glucose tolerance test or glycosylated haemoglobin C ≥ 6.5%), non-alcoholic fatty liver disease (NAFLD, confirmed by ultrasound or computed tomography), and medication history (drugs used over 4 months in the past year).

A modified Food Frequency Questionnaire (FFQ) was used to collect information on participants’ diets. Daily milk consumption was recorded. Milk types included plain milk (ultra-heated sterilized plain milk or pasteurized milk), reconstituted milk, flavoured milk, or fermented milk (with a content of ≥80% cow’s milk or milk powder), measured in millilitres (ml). In this study, the main types of milk consumed by the individuals were ultra-heated sterilized plain milk, pasteurized milk, and fermented milk.

Blood samples were collected after 8 h of fasting. The samples were centrifuged at 3000 rpm at room temperature for 10 min; the supernatants were collected and stored in aliquots at −80 °C until use for routine and biochemical tests.

### 2.2. Sample Collection

Faecal samples were collected in sterile tubes containing a DNA stabiliser (Invitek, Berlin, Germany) according to the field sampling protocol. The samples were stored at −80 °C for further analysis. Total bacterial genomic DNA was extracted from the faecal samples using a TIANamp Bacteria DNA Kit (TIANGEN, Beijing, China). The eligibility criteria for faecal sampling were as follows: (1) no treatment with antibiotics or probiotics within 1 month before sample collection; and (2) no diarrhoea, fever, or infection within 1 month of sample collection.

### 2.3. Sequencing and Bioinformatics

A DNA library was constructed using the TruSeq Nano DNA LT Library Preparation Kit (FC-121-4001) (Illumina, San Diego, CA, USA). DNA was fragmented using dsDNA fragmentase (M0348S; NEB) at 37 °C for 30 min. Library construction began with fragmented cDNA. Blunt-end DNA fragments were generated using a combination of fill-in reactions and exonuclease activity, and size selection was performed using the provided sample purification beads. An A-base was then added to the blunt ends of each strand to prepare them for ligation to indexed adapters. Each adapter contained a T-base overhang for ligation to the A-tailed fragmented DNA. These adapters contained the full complement of the sequencing primer hybridisation sites for single-indexed, paired-end, and indexed reads. Single- or dual-index adapters were ligated to the fragments, and the ligated products were amplified with PCR under the following conditions: initial denaturation at 95 °C for 3 min; 8 cycles of denaturation at 98 °C for 15 s; annealing at 60 °C for 15 s; extension at 72 °C for 30 s; and then final extension at 72 °C for 5 min. We conducted paired-end sequencing (2 × 150) using the Illumina Hiseq 2000 sequencing platform (Illumina, San Diego, CA, USA).

The raw sequencing reads were processed to obtain valid reads for further analysis. First, sequencing adapters were removed from the sequencing reads using Cutadapt v1.9 [20]. Second, low-quality reads were trimmed using fqtrim v0.94, a sliding-window algorithm. Third, reads were aligned to the host genome using Bowtie2 v2.2.0 to remove host contamination. Once quality-filtered reads were obtained, they were assembled de novo to construct the metagenome of each sample using IDBA-UD v1.1.1 [21]. All coding regions (CDSs) of the metagenomic contigs were predicted using MetaGeneMark v3.26 [22]. The CDS sequences of all samples were clustered using CD-HIT v4.6.1 [23] to obtain unigenes. The unigene abundance of each sample was estimated by TPM based on the number of aligned reads using Bowtie2 v2.2.0 [24]. The lowest common ancestor taxonomy of the unigenes was obtained by alignment against the NCBI NR database using DIAMOND v0.9.14 [25]. Based on the taxonomic and functional annotation of unigenes, along with the abundance profile of unigenes, differential analysis was carried out at each taxonomic, functional, or gene level using Fisher’s exact test (categorical variables) or the Kruskal–Wallis test (continuous variable analysis). We used the linear discriminant analysis (LDA) effect size (LEfSe) [26] method to identify species with statistically significant differential abundance among groups, and results were validated with ALDEx2 (Table S1) [27]. Multivariate Association with Linear Models (MaAsLin) [28] was used to analyse the relationship between gut microbiota and longevity. The vegan (v 2.5-7) package [29] was used for calculating alpha diversity (Shannon’s and Simpson’s indexes) with bacterial relative abundance at the species level. The beta-diversity was calculated with the constrained principal coordinate analysis based on the Bray–Curtis distance [30].

### 2.4. Longevity-Associated Microbial Index

The longevity-associated microbial index (LAMI) was defined as the log value of the total abundance of bacterial genera enriched in the “longe” group (aged 90–98) divided by the total abundance of bacterial genera depleted in the “longe” group [31,32,33], which was expressed as follows:

$$LAMI=\log_{2}\frac{\Sigma_{e}}{\Sigma_{d}}$$

where “*e*” presented bacterial contents enriched in the “longe”, and “*d*” presented the depleted. Based on bacterial contents with significant differences between the “longe” and the “CON” groups (aged 60–89) at different levels (genus, species, and strain), three LAMIs were calculated and used for describing the profiles of the bacterial community in long-lived individuals. At the genus level, *Akkermansia* was enriched in the “longe” group, and *Alcaligenes*, *Candidatus_Saccharibacteria_noname_unclassified*, *Clostridiales_Family_XIII_Incertae_Sedis_noname*, *Granulicatella*, and *Weissella* were depleted. At the species level, *Akkermansia muciniphila*, *Bifidobacterium adolescentis*, *Lactobacillus salivarius*, *Clostridium bartlettii*, and *Streptococcus anginosus* were enriched in the “longe” group, and *Alcaligenes_unclassified*, *Bacteroides_sp_3_1_19*, *Bifidobacterium dentium*, *Eubacterium infirmum*, *Lachnospiraceae bacterium_1_4_56FAA*, and *Streptococcus australis* were depleted. At the strain level, *GCF_000020225*, *GCF_000154445*, *Bifidobacterium_dentium_unclassified*, *Lactobacillus_salivarius_unclassified*, and *Streptococcus_anginosus_unclassified* were enriched, while *Bifidobacterium_adolescentis_unclassified*, *GCF_000163655*, *GCF_000218385*, *GCF_000242675*, and *Streptococcus_australis_unclassified* were depleted in the “longe” participants.

### 2.5. Functional Profiles of Microbial Communities with HMP Unified Metabolic Analysis Network 2 (HUMAnN2)

The HMP Unified Metabolic Analysis Network 2 (HUMAnN2) is a tiered search strategy that enables fast, accurate, and species-resolved functional profiling of host-associated and environmental communities [34,35]. To investigate the role of microbes in promoting longevity, we profiled the functions of HUMAnN2-based microbial genes.

### 2.6. Statistical Analysis

R software (v4.0.1; R Foundation for Statistical Computing, Vienna, Austria) with ggplot2 (v3.3.2) and pheatmap packages was used for data visualisation. SPSS software (version 20.0; IBM, Armonk, NY, USA) was used for statistical analysis, and numerical variables are expressed as mean values ± standard deviation (*x ± s*). An analysis of variance was used to compare the variables between the groups, and the chi-square test was used to analyse categorical variables among the groups. Multiple testing was used with the Bonferroni test. All statistical results were significant when the *p*-value ≤ 0.05 (two-tailed). The *p*-value was corrected with FDR, and FDR < 0.05 was considered significant.

## 3. Results

### 3.1. Demographic and Clinical Data of the Study Population

**Figure 1:**
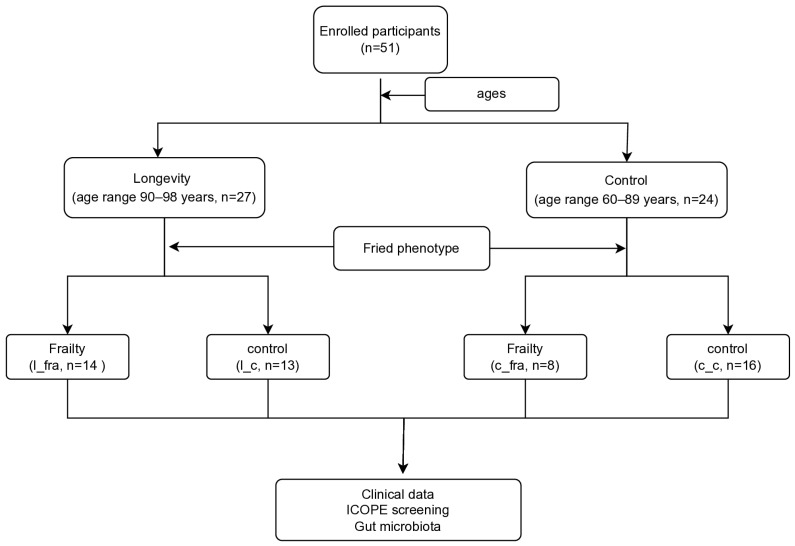
Flowchart of study. ICOPE: Integrated Care for Older People tool.

A total of 27 long-lived adults (longe group, age range 90–98 years, labelled as “longe”) and 24 young-old adults (control group, age range 60–89 years, labelled as “CON”) were enrolled in this study. The mean ages (SD) were 91.6 (1.9) years and 79.1 (6.8) years, respectively. The female population was higher in the CON group than in the longe group (58.3% vs. 29.6%, *p* = 0.039). No significant differences were observed between the longe and CON groups regarding DM, HTN, coronary heart disease (CHD), and NAFLD rates. In the longe group, 14 patients (51.9%) were defined as frail based on the FP criteria. Eight patients (33.3%) in the CON group were considered frail (Figure 1). The prevalence of impaired intrinsic capacity was 92.6% and 62.5% in the longe and CON groups, respectively. The longe group had lower scores for cognitive function (MMSE) (*p* = 0.001), nutritional status (MNA_SF) (*p* < 0.001), osteoporosis (osteoporosis self-assessment tool for Asians, OSTA) (*p* = 0.004), and intrinsic capacity (*p* = 0.017) (Table 2). Furthermore, the longe group had lower serum albumin levels and higher creatinine levels (both *p* < 0.05). Multifactorial analysis showed that higher daily milk intake (300–500 mL/d vs. <300 mL/d) and higher MNA-SF scores were independent protective factors against frailty, while ageing was an independent risk factor (Figure S3C).

### 3.2. Bacterial Diversity, Abundance, and Functionality

**Figure 2:**
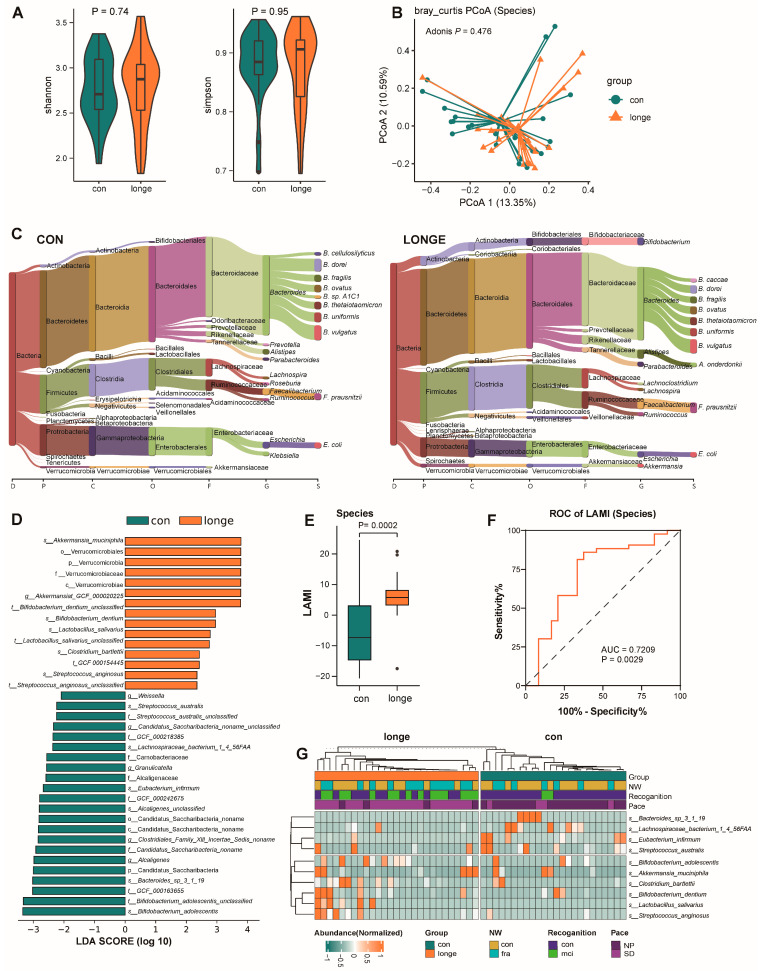
The characteristics of gut microbiota in long-lived (longe) and young-old (CON) adults. (**A**) The microbiota’s alpha diversity is assessed using Shannon and simpson indices. In the box plots, the horizontal lines indicate the median values. The upper and lower boundaries of the boxes correspond to the 75th and 25th percentiles, respectively. (**B**) Principal coordinate analysis based on Bray-Curtis dissimilarity at species level. R software (v 4.0.1) with the vegan (v 2.5-7) package were used for analysis. *p*-values were acquired using permutational multivariate analysis of variance (PERMANOVA). (**C**) The characteristics of microbial communities in two groups of people at various taxonomic levels (domain, phylum, class, order, family, genus, and species). (**D**) The microbiota with the most significant differences between long-lived and young-old group. (**E**) LAMI of longe and CON group. (**F**) The efficiency of LAMI in distinguishing between long-lived and young-old group. (**G**) Heatmap of bacterial abundance with significant difference in different groups. Abbreviations: LAMI: The longevity-associated microbial index, MCI: mild cognitive impairment, NP: normal pace, SD: gait speed decreased.

No significant differences were observed in Shannon’s index, Simpson’s index, or the principal component analysis between the longe (aged 90–98 years) and CON groups (aged 60–89 years) (all *p* > 0.05). The bacterial composition between the two groups at the phylum to species level is shown in Figure 2 and Figure S1C–I. Further analyses of the gut microbiota structure revealed significant changes in Verrucomicrobia at the phylum level in the longe group. This trend was similar at the bacterial order (Verrucomicrobiales), family (Verrucomicrobiaceae), and species (*Akkermansia mucinilhilla*) level (Figure 2A,B and Figure S1A,B). In addition, the longe group had a higher abundance of *Bifidobacterium dentium* at the species level. Furthermore, *Lactobacillus salivarius*, *Clostridium bartiletti*, and *Streptococcus anginosus* were more abundant at the species level in the longe group (Figure 2C,D). Notably, *Klebsiella*, *Roseburia*, and *Prevotellas* at the genus level and *Streptococcus austrails*, *Eubacterium infirmum*, *Bacteroides*_*sp*_3_1_19, and *Bifidobacterium adolescentis* at the species level declined significantly in the longe group (Figure 2C,D). The LAMI values were calculated based on the bacterial abundance at the genus, species, and strain levels (Figure 2E and Figure S1). The data showed that the differences in LAMI values were most pronounced at the species level between the longe and CON groups (*p* = 0.0002; Figure 2E). Additionally, we tested the differential efficiency of LAMI using a receiver operating characteristic (ROC) curve. The area under the curve (AUC) was 0.7209 (*p* = 0.0029; Figure 2F). At the genus and strain levels, the differences were significant (*p* = 0.0013 and 0.003, respectively) (Figure S1J–M).

**Figure 3:**
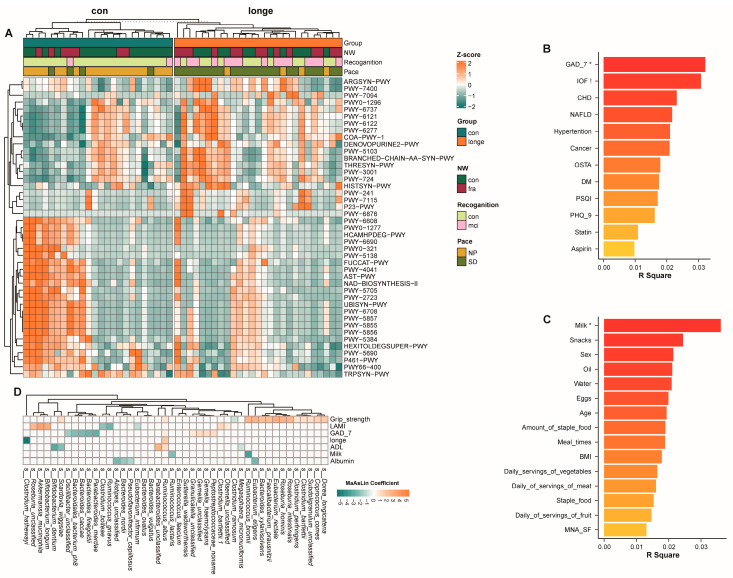
(**A**) Functional pathways in long-lived and young-old adults. (**B**–**D**) Related dietary and clinical factors and the association between gut microbiota and clinical assessments. (**B**,**C**) R software (v 4.0.1) with the vegan (v 2.5-7) package were used for analysis. *p*-values were acquired using permutational multivariate analysis of variance (PERMANOVA). *: FDR < 0.05, !: 0.05 < FDR < 0.1. Abbreviations: GAD-7: Brief 7-Item Self-Report Questionnaire for Generalised Anxiety Disorder, IOF: one-minute osteoporosis risk test, CHD: chronic heart disease, NAFLD: non-alcoholic fatty liver disease, OSTA: osteoporosis self-assessment tool for Asians, DM: diabetes mellitus, PSQI: Pittsburgh Sleep Quality Index, PHQ-9: Patient Health Questionnaire-9, ADL: activity of daily living, BMI: body mass index, MNA_SF: Mini Nutritional Assessment-Short Form, MCI: mild cognitive impairment, NP: normal pace, SD: gait speed decreased.

Having identified the aforementioned differences in microbial diversity and structure, we further analysed the association between microbiota and clinical assessment results for frailty and intrinsic capacity. We detected a significant association between *Akkermansia mucinilhilla* and frailty, recognition, and gait speed (Figure 2G). Predictive functional analysis revealed differences in functional pathways. Pathways related to protein processing, nutrient metabolism, and cellular renewal, including L-isoleucine biosynthesis III, the super-pathway of branched amino acid biosynthesis, L-arginine biosynthesis, and starch degradation, were overrepresented in the longe group (Figure 3 and Figure S2A). *Faecalibacterium prausnitzii* and *Escherichia coli* contributed most to the branched amino acid biosynthesis pathway and L-arginine biosynthesis I, respectively (Figure 3A and Figure S2B–I). We further analysed the association between diet, clinical scores, and gut microbiota composition and function. GAD-7 scores in clinical data and daily milk consumption had the highest relevance. We also analysed the relationship between gut microbiota and grip strength by MaAsLin coefficient (Figure 3B,C). GAD-7 scores were negatively correlated with *Parabacteroides merdae* (coefficient = −2.81, *p* = 0.004), *Bacteroides finegoldii* (coefficient = −2.57, *p* = 0.012), and *Bacteroides cacae* (coefficient = −2.30, *p* = 0.014) and positively correlated with *Peptostreptococcaceae noname* (coefficient = 1.19, *p* =0.002) and *Gemella haemolysans* (coefficient = 1.19, *p* = 0.017). Daily milk consumption was negatively correlated with *Ruminosoccus bromii* (coefficient = −3.50, *p* = 0.005) and *Ruminosoccus lactaris* (coefficient =−2.11, *p* = 0.001). Grip strength was positively correlated with *Roseburia hominis* (coefficient = 2.63, *p* < 0.001), *Eubacterium rectale* (coefficient = 2.94, *p* = 0.005), *Eubacterium eligens* (coefficient = 2.72, *p* = 0.003), and *Roseburia intestinalis* (coefficient = 2.56, *p* = 0.006) and negatively correlated with *Clostridium ramosum* (coefficient = −0.87, *p* = 0.015) (Figure 3D and Figure S3).

### 3.3. Gut Microbiota and Clinical Data Based on Age and Frailty

To explore the correlation between the gut microbiota, longevity, and physical function, the participants were divided into four groups based on age and the presence of frailty: long-lived adults with frailty (l_fra, *n* = 14), long-lived adults without frailty (l_c, *n* = 13), young-old adults with frailty (c_fra, *n* = 8), and young-old adults without frailty (c_c, *n* = 16) (Figure 1).

**Figure 4:**
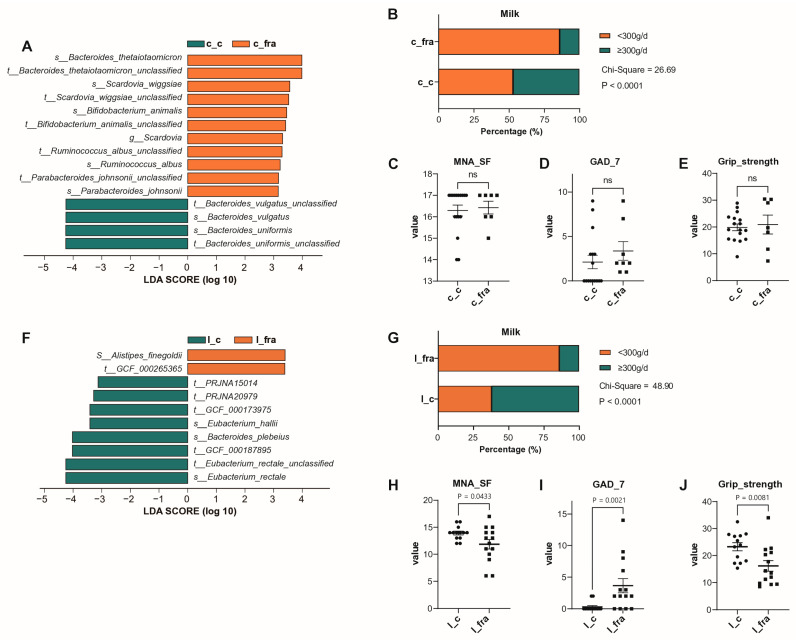
Comparison of gut microbiota, clinical assessments and daily milk intake in different subgroups. (**A**–**E**): c_c versus c_fra, (**F**–**J**): l_c versus l_fra). Abbreviations: l_fra: frail nonagenarian individuals (aged 90–98), l_c: non-frail individuals aged 90–98, c_fra: frail individuals aged 60–89, c_c: non-frail individuals aged 60–89, MNA_SF: Mini Nutritional Assessment-Short Form, GAD-7: Brief 7-Item Self-Report Questionnaire for Generalised Anxiety Disorder, ns: no significance.

Compared to the l_c group, the l_fra group had lower cognition (MMSE) and nutritional state (MNA_SF) scores, more depression (PHQ-9), and worse sleep (Pittsburgh Sleep Quality Index, PSQI) (all *p* < 0.05). The proportion of adults who consumed 300–500 mL of milk per day was significantly higher in the l_c group (*p* < 0.01, Figure 4F–J). *Eubacterium hallii* and *Bacteroides plebeius* were more abundant in the l_c group, whereas *Alistipes finegoldii* was more abundant in the l_fra group (Figure 4A,F).

When c_c and c_fra were compared, the percentage of adults who consumed more than 300–500 mL of milk was significantly lower in c_fra. No significant differences were found in MNA_SF, GAD_7, or grip-strength scores between the c_c and c_fra groups (all *p* > 0.05). *Bacteroides thetaiotaomicron*, *Scardovia wiggsiae*, and *Ruminococcus albus* were more abundant in the c_fra group, whereas *Bacteroides vulgatus* and *Bacteroides uniformis* were more abundant in the c_c group (Figure 4A–E).

**Figure 5:**
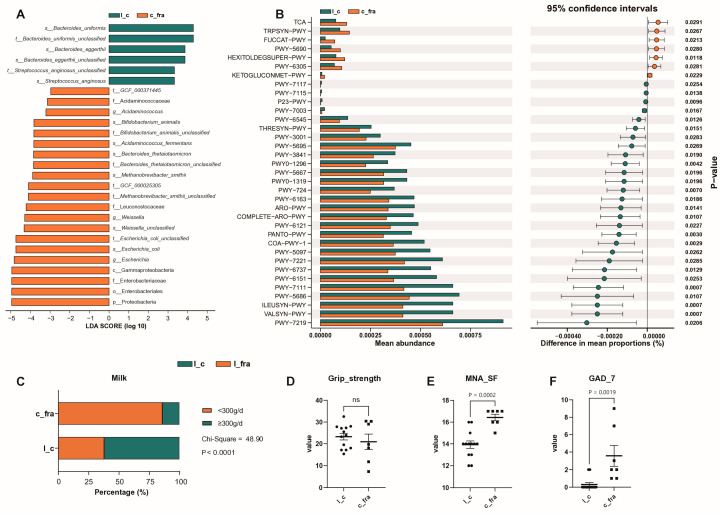
The comparative analysis of gut microbiota, functional pathways, and clinical data between l_c and c_fra groups. (**A**): The microbiota with the most significant differences between l_c and c_fra groups. (**B**): Functional pathways in l_c and c_fra groups. (**C**−**F**): Clinical assessments and daily milk intake in l_c and c_fra groups. Abbreviations: l_fra: frail nonagenarian individuals (aged 90–98), l_c: non-frail individuals aged 90–98, c_fra: frail individuals aged 60–89, c_c: non-frail individuals aged 60–89, MNA_SF: Mini Nutritional Assessment-Short Form, GAD-7: Brief 7-Item Self-Report Questionnaire for Generalised Anxiety Disorder, ns: no significance.

The c_fra group was younger but had worse physical function, whereas the l_c group had longevity with preserved physical function. These two groups differed the most when age and physical function were taken into account. We analysed dietary habit, bacterial structure, and functional pathways of the l_c and c_fra groups (Figure 5 and Figure S4). *Bacteroides uniformis*, *Bacteroides eggerthii,* and *Streptococcus anginosus* increased at the species level in the l_c group. C_fra showed higher levels of *Escherichia coli*, *Bifidobacterium animalis*, *Acidaminococus fermentans*, and *Bacteroides thetaiotaomicron* (Figure 5A). Many pathways were overrepresented in the l_c group compared with c_fra group, including chorismate biosynthesis; aromatic amino acid biosynthesis; biosynthesis of L-isoleucine, L-lysine, CDP-diacylglycerol, S-adenosyl-L-methionine, L-threonine, L-methionine, and L-valine; de novo biosynthesis of adenosine ribonucleotides; and de novo biosynthesis of guanosine ribonucleotides. Underrepresented pathways in the l_c group included fucose degradation, hexitol degradation in the TCA cycle, and ketogluconate metabolism (Figure 5B). The percentage of subjects who consumed more than 300 mL/d of milk was significantly higher in the l_c group (Figure 5C). The c_fra group had higher GAD_7 scores and MNA_SF, while there was no significant difference in grip strength between the l_c and c_fra group (Figure 5D–F).

## 4. Discussion

With the rapid ageing of the world’s population and the desire for healthy ageing, age-related issues are receiving increasing attention. Our results showed that impaired intrinsic capacity and frailty prevalence were higher in long-lived adults. The intrinsic capacity scores of long-lived adults were significantly lower than those of young-old adults. In the longe group, no significant differences were found in albumin, haemoglobin, creatinine, erythrocyte sedimentation rate (ESR), and C-reactive protein (CRP) levels between the frail (l_fra) and control (l_c) adults, while the l_fra group had lower MNA_SF scores and higher PHQ-9 and PSQI scores. Long-lived adults with frailty had worse nutritional and psychological states. Clinical assessment scales combining subjective and objective factors may be more sensitive than single clinical indicators in identifying long-lived adults with impaired physical function. Multifactorial analysis showed that ageing, poor nutritional status (lower MNA_SF score), and lower daily milk consumption (300–500 mL/d vs. <300 mL/d) were independently associated with frailty in older adults. These results confirm the importance of intrinsic capacity for the long-lived adults [36], suggesting the positive role of good nutrition, emotions, and sleep status. The correlation between gut microbiota and the clinical assessment scales deserves further exploration.

A previous study showed that chao1, observed species, and Shannon index increased with age after 60, peaked at 90, and declined thereafter [37]. This study observed no significant decline in long-lived adults compared with the young-old group in overall taxonomic diversity. Nevertheless, the composition of the bacteria differs. *Akkermansia muciniphila* and *Bifidobacterium dentium*, known as health-associated genera, increased in the long-lived adults. Meanwhile, *Akkermansia muciniphila* was positively correlated with LAMI. *Akkermansia muciniphila* modulates energy metabolism and glucose tolerance, promotes immunomodulation, and promotes metabolic homeostasis. Thus, *Akkermansia muciniphila* may be a next-generation probiotic [38,39]. This correlates with a previous study [6]. Functional analysis suggests *Akkermansia muciniphila* is involved in the super-pathway of branched amino acid (BCAA) biosynthesis and 5-aminoimidazole ribonucleotide biosynthesis I. BCAA is a substrate for nitrogenous compound synthesis and regulates the metabolism of glucose, lipid and protein synthesis, intestinal health, and immunity via a special signal network, including phosphoinositide 3-kinase/protein kinase B/mammalian target of rapamycin (PI3K/AKT/mTOR) [40]. In the functional pathways analysis, amino acid synthesis pathways were overrepresented in the l_c group compared to the c_fra group. Enrichment of this bacterium and altered pathways in long-lived adults with better physical function indicate that *Akkermansia muciniphila*, protein synthesis, and metabolism are closely associated with healthy ageing. In the subgroup analysis, compared to the l_fra group, the l_c group was enriched in *Bacteroides plebeius*. *Bacteroides plebeius* has shown the potential to combat muscle atrophy via the Mystn/ActRIIB/SMAD3 pathway, enhancing the barrier function of the intestinal mucosa and reducing protein consumption in animal studies [41]. In long-lived adults, changes in gut microbiota are associated with physical function.

The l_c (long-lived adults without frailty) and c_fra (young-old adults with frailty) groups had the most remarkable difference in combined age and physical function scores. We expected that a comparison of these two groups might identify changes in gut microbiota and function that are most closely related to healthy aging. Those in the C_fra group were not inferior to those in the l_c group in terms of cognitive function, gait speed, and MNA_SF. While the l_c group showed an increased abundance of *Bacteroides eggerthii*, the c_fra group showed a higher abundance of *Escherichia coli. Bacteroides eggerthii* has been shown to produce short-chain fatty acids (SCFAs) that can regulate intestinal immunity and inflammation and promote a healthy intestinal environment [42,43]. No differences in SCFA pathway were observed in this study. *Escherichia coli* is a well-known opportunistic pathogen [44]. The results suggest that some beneficial bacteria are enriched in long-lived adults, especially those with preserved physical function, whereas opportunistic pathogenic bacteria are enriched in frail older adults. Changes in gut microbiota may precede clinical indicators.

In the subsequent analysis, we analysed the relationship between gut microbiota and clinical indicators such as grip strength and gait speed, as well as possible related functional pathways. Grip strength was positively correlated with *Roseburia hominis*, which can produce butyrate and thus alleviate neuroinflammation [45,46]. No studies have been reported on the relationship between grip strength and *Roseburia hominis*. Based on the characteristics of *Roseburia hominis*, we speculated that it may affect muscle function, and furthermore gait speed, by acting on short-chain fatty acid metabolism and gut immune pathways. Considering that this study is a cross-sectional observational study with a limited sample size, the association and hypothesis need further verification. Studies have found that animal-based diets decrease the levels of Firmicutes (including *Ruminococcus bromii*), which metabolise dietary plant polysaccharides [47]. This study showed that milk consumption was negatively correlated with the abundance of *Ruminococcus bromii.* This is consistent with the results of previous studies. The affected pathways included the synthesis of L-lysine, L-threonine, and L-methionine amino acids. Diets may impact physical function by affecting the gut microbiota and functional pathways.

This study’s findings are limited, as we only evaluated the correlation between microbiota and physical function in a cross-sectional study and conducted preliminary explorations. Furthermore, it is impossible to determine whether the altered bacteria and pathways are temporary or long-lasting. The causal relationship between the gut microbiota and physical function is unclear. In addition, collecting food intake information was challenging because it was difficult to accurately record the daily dietary consumption of participants aged over 90 years. Some consumed nutrients were also calculated. The mechanisms underlying the association between the microbiota and physical function should be confirmed by cohort studies with large samples and long-term follow-up.

## 5. Conclusions

In conclusion, this study found no decline in the abundance of gut microbiota in long-lived individuals. Bacteria of long-lived adults were enriched in *Akkermansia muciniphila*, *Bifidobacterium dentium*, and *Bacteroides plebeius*, which may have beneficial effect for health. Furthermore, gut microbiota have a close relationship with physical function and intrinsic capacity. Possibly related pathways included amino acid synthesis and SCFA metabolism. During the process of increasing frailty, changes in gut microbiota may precede clinical indicators. Thus, gut microbiota may serve as a potential biological marker of healthy ageing.

## Figures and Tables

**Table 1:** Five domains constituting the intrinsic capacity measured by the WHO ICOPE screening tool.

Intrinsic Capacity	Tests	Scores
Cognition	MMSE	Scores equal or greater than 27 (1)/less than 27 (0)
Locomotion Chair rise test	Rise from a chair five times without using arms within 14 s	Yes (1)/No (0)
Vitality		
Weight loss	Have you unintentionally lost more than 3 kg over the last 3 months?	Yes (0)/No (1)
Appetite loss	Have you experienced loss of appetite?	Yes (0)/No (1)
Sensory		
Visual impairment	Do you have any problems with your eyes: difficulties in seeing far, reading, or eye disease?	Yes (0)/No (1)
Hearing loss	Do you have any problems in hearing: difficulties in hearing whispers?	Yes (0)/No (1)
Psychosocial		
Depressive symptoms	PHQ-9 score	Scores greater than 4 (0)/scores equal to or less than 4 (1)
Anxiety symptoms	GAD-7 score	Scores greater than 4 (0)/scores equal to or less than 4 (1)

Abbreviations: MMSE: Mini-Mental State Examination, PHQ-9: Patient Health Questionnaire-9, GAD-7: Brief 7-Item Self-Report Questionnaire for Generalised Anxiety Disorder.

**Table 2:** Demographic and clinical data of long-lived and young-old adults.

	Longe Group vs. CON Group			Longe Group			CON Group		
	Longe Group (≥90, *n* = 27)	CON Group (60–89, *n* = 24)	*p* Value	l_fra (*n* = 14)	l_c (*n* = 13)	*p*	C_fra (*n* = 8)	C-c (*n* = 16)	*p* Value
Age	91.59 ± 1.89	79.08 ± 6.83	<0.001	91.85 ± 1.66	91.30 ± 2.14	0.406	80.75 ± 5.65	78.25 ± 7.37	0.410
Female	8 (29.6%)	14 (58.3%)	0.039	5 (35.7%)	3 (23.1%)	0.678	1 (12.5%)	13(81.2%)	0.002
DM	6 (22.2%)	10 (41.7%)	0.135	3 (21.4%)	3 (23.1%)	1.000	6 (75.0%)	4(25.0%)	0.032
HTN	18 (66.7%)	18 (75.0%)	0.514	11 (78.6%)	7 (53.8%)	0.236	7 (87.5%)	11(68.8%)	0.621
CHD	8 (30.8%)	4 (16.7%)	0.243	6 (42.9%)	2 (16.7)	0.216	3 (37.5%)	1(6.2%)	0.091
NAFLD	2 (7.4%)	6 (25.0%)	0.127	1 (7.1%)	1 (7.1%)	1.000	1 (12.5%)	5(31.2%)	0.621
Statin	14 (51.9%)	16 (66.7%)	0.283	11 (78.6%)	3 (23.1%)	0.004	6 (75.0%)	10(62.5%)	0.667
Metformin	0 (0.0%)	5 (20.8%)	0.018	-	-	-	3 (37.5%)	2(12.5%)	0.289
Aspirin	8 (29.6%)	9 (37.5%)	0.552	6 (42.9%)	2 (15.4%)	0.209	4 (50.0%)	5(31.2%)	0.412
Albumin	35.36 ± 3.65	39.06 ± 4.09	0.008	34.74 ± 3.85	37.83 ± 0.40	0.200	36.90 ± 4.93	40.29 ± 3.06	0.059
Haemoglobin	121.40 ± 19.48	126.27 ± 17.33	0.430	118.83 ± 19.93	131.67 ± 16.56	0.325	124.00 ± 28.33	127.57 ± 6.92	0.736
BMI	24.70 ± 3.50	24.12 ± 3.09	0.537	25.25 ± 4.38	24.10 ± 2.21	0.406	23.82 ± 2.52	24.27 ± 3.40	0.747
Grip strength	19.56 ± 7.27	20.17 ± 6.44	0.756	15.00 ± 5.21	24.48 ± 5.88	<0.001	19.41 ± 9.64	20.54 ± 4.45	0.760
Time_4.57m	10.02 ± 5.66	4.80 ± 2.45	<0.001	6.61 ± 3.62	13.18 ± 5.43	0.001	6.33 ± 3.70	4.04 ± 0.97	0.126
CRP	8.82 ± 18.42	3.68 ± 9.19	0.331	10.80 ± 20.26	0.90 ± 0.46	0.425	6.69 ± 15.21	1.96 ± 1.76	0.410
ESR	15.80 ± 17.22	16.87 ± 24.06	0.883	18.42 ± 18.40	5.33 ± 3.06	0.254	26.50 ± 36.96	11.36 ± 10.48	0.292
Creatinine	95.33 ± 29.93	76.50 ± 18.31	0.023	96.50 ± 33.44	90.67 ± 9.02	0.775	84.87 ± 17.33	71.71 ± 17.67	0.106
BUN	6.50 ± 2.50	7.07 ± 1.70	0.404	6.59 ± 2.77	6.14 ± 1.15	0.794	7.15 ± 1.66	7.04 ± 1.78	0.882
MMSE	24.78 ± 4.50	28.17 ± 1.20	0.001	22.57 ± 4.78	27.15 ± 2.70	0.006	27.62 ± 1.06	28.44 ± 1.21	0.121
MNA-SF	12.85 ± 2.68	16.33 ± 0.96	<0.001	11.50 ± 2.93	14.31 ± 1.38	0.004	16.50 ± 0.76	16.25 ± 1.06	0.561
GAD-7	2.04 ± 3.43	2.54 ± 3.02	0.582	3.21 ± 4.21	0.77 ± 1.74	0.061	3.13 ± 3.18	2.25 ± 3.00	0.516
PHQ-9	2.67 ± 3.82	3.13 ± 3.89	0.674	4.36 ± 4.40	0.85 ± 1.95	0.014	4.13 ± 3.80	2.63 ± 3.96	0.385
PSQI	8.67 ± 4.22	9.12 ± 3.65	0.682	11.29 ± 3.73	5.85 ± 2.64	<0.010	10.00 ± 3.70	8.69 ± 3.66	0.419
OSTA	−5.32 ± 2.27	−3.42 ± 2.18	0.004	−5.19 ± 2.69	−5.47 ± 1.81	0.754	−3.14 ± 2.68	−3.56 ± 1.96	0.662
Intrinsic capacity	5.44 ± 2.15	6.71 ± 1.46	0.017	4.00 ± 1.66	7.00 ± 1.41	<0.010	7.15 ± 1.04	4.50±1.29	<0.010

Abbreviations: l_fra: frail nonagenarian individuals (aged 90–98), l_c: non-frail individuals aged 90–98, c_fra: frail individuals aged 60–89, c_c: non-frail individuals aged 60–89, DM: diabetes mellitus, HTN: hypertension, CHD: chronic heart disease, NAFLD: non-alcoholic fatty liver disease, BMI: body mass index, CRP: C-reactive protein, ESR: erythrocyte sedimentation rate, BUN: blood urea nitrogen, MMSE: Mini-Mental State Examination, MNA-SF: Mini-Nutritional Assessment Short Form, PHQ-9: Patient Health Questionnaire-9, GAD-7: Brief 7-Item Self-Report Questionnaire for Generalised Anxiety Disorder, OSTA: osteoporosis self-assessment tool for Asians.

## Data Availability

The original data presented in the study are openly available in the Genome Sequence Archive (Genomics, Proteomics & Bioinformatics 2021) in the National Genomics Data Center (Nucleic Acids Res 2022), China National Center for Bioinformation/Beijing Institute of Genomics, Chinese Academy of Sciences (GSA: CRA012774) at https://ngdc.cncb.ac.cn/gsa (accessed on 21 September 2024).

## Supplementary Materials

The following supporting information can be downloaded at: https://www.mdpi.com/article/10.3390/microorganisms13071657/s1, Figure S1. Details of gut microbiota in long-lived and young-old adults. (A,B) Principal coordinate analysis based on Bray-Curtis dissimilarity at genus and strain levels. (J−M) The efficiency of LAMI in distinguishing between long-lived and young-old group at genus and strain level. Figure S2. Altered pathways in the longe and CON groups and major contributing bacteria. (A) Functional pathway in longe and CON groups, (B−I) Microbial contributions to different functional pathways. Figure S3. Association between clinical factors, frailty, and gut microbiota. (A,B) R software (v 4.0.1) with the vegan (v 2.5-7) package were used for analysis. (C) Clinical indicators associated with frailty; (D) Microbial abundance in adults with different daily milk intakes. (E−H) The correlation between microbiota and clinical indicators, as well as LAMI. Figure S4. Altered pathways in subgroups. (A) Functional pathway in c_c and c_fra group, (B) Functional pathway in l_c and l_fra group. Figure S5. Flowchart of study. Table S1. Results of differential abundance analysis by ALDEx2.

## Abbreviations

DM	diabetes mellitus
WHO	World Health Organisation
ICOPE	Integrated Care for Older People
MMSE	Mini-Mental State Examination
SPPB	Short Physical Performance Battery
MNA_SF	Mini-Nutritional Assessment Short Form
GAD-7	Brief 7-Item Self-Report Questionnaire for Generalised Anxiety Disorder
PHQ-9	Patient Health Questionnaire-9
MCI	mild cognitive impairment
IC	impaired intrinsic ability
NAFLD	non-alcoholic fatty liver disease
HTN	hypertension
FFQ	Food Frequency Questionnaire
LAMI	The longevity-associated microbial index
CHD	coronary heart disease
BCAA	branched amino acid
