# Supplementary material for: Healthy Ageing and Gut Microbiota: A Study on Longevity in Adults
Source: Microorganisms. 2025 Jul 14;13(7):1657. doi: 10.3390/microorganisms13071657 (PMC12298205; doi:10.3390/microorganisms13071657)

Supplementary materials

Healthy ageing and gut microbiota: a study on longevity in adults

Lihua Deng ^1#^, M.D.; Jun Xu ^2,3#^, Ph.D.; Qian Xue ^1^, M.D.; Wei Yanan, M.D. ^1^; Jingtong Wang ^1^* and M.D.

^1^ Department of Geriatrics, Peking University People's Hospital, Beijing, China

^2^ Department of Gastroenterology, Peking University People's Hospital, Beijing, China

^3^ Clinical Center of Immune-Mediated Digestive Diseases, Peking University People's Hospital, Beijing, China

^*^ Correspondence to Jingtong Wang (E-mail: wangjingtong@pkuph.edu.cn), Department of Geriatrics, Peking University People's Hospital, No. 11 Xizhimen South Street, Xicheng District, Beijing 100044, P. R. China.

^#^ These authors contributed equally to the study.

**Figure S1.** Details of gut microbiota in long-lived and young-old adults.


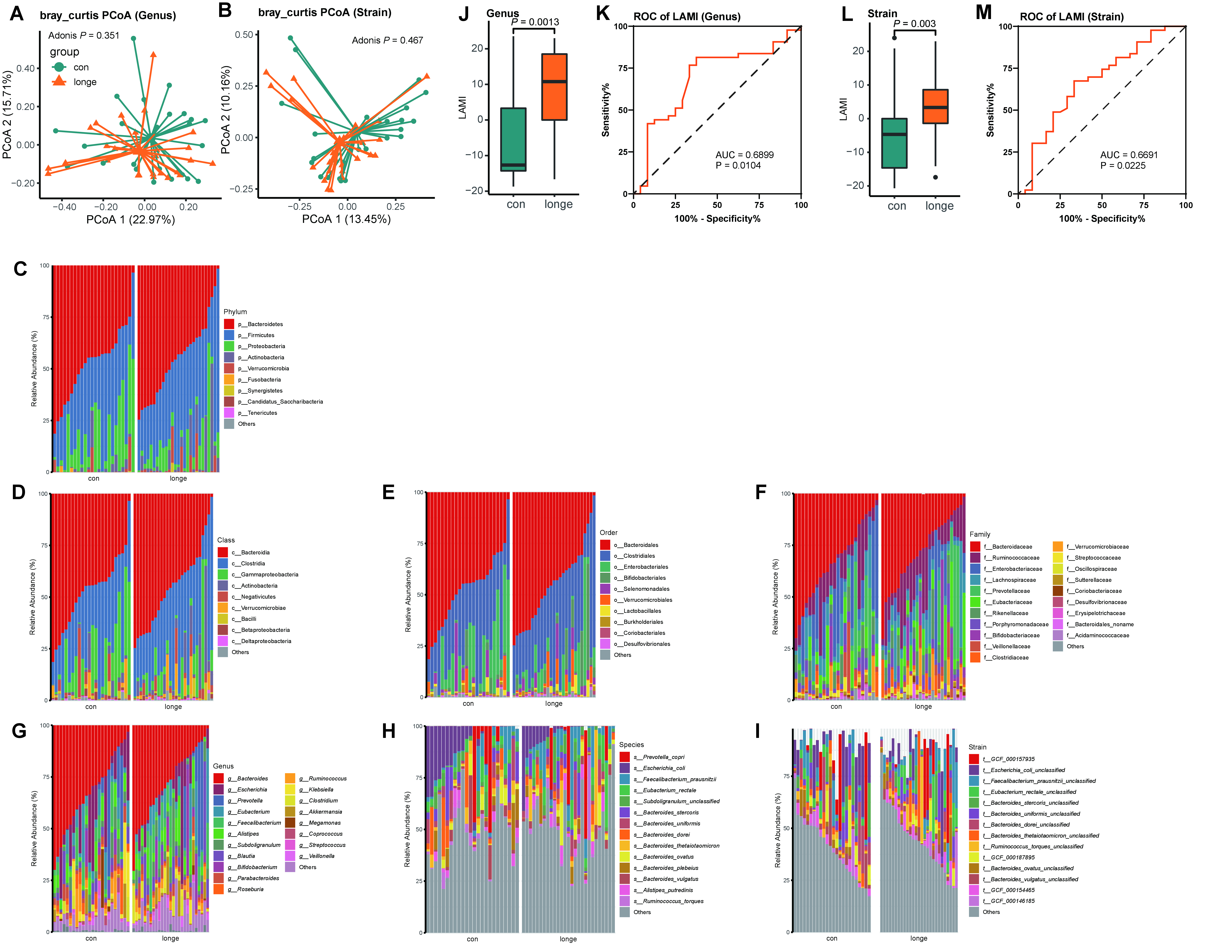


**Figure S2.** Altered pathways in the longe and CON groups and major contributing bacteria.


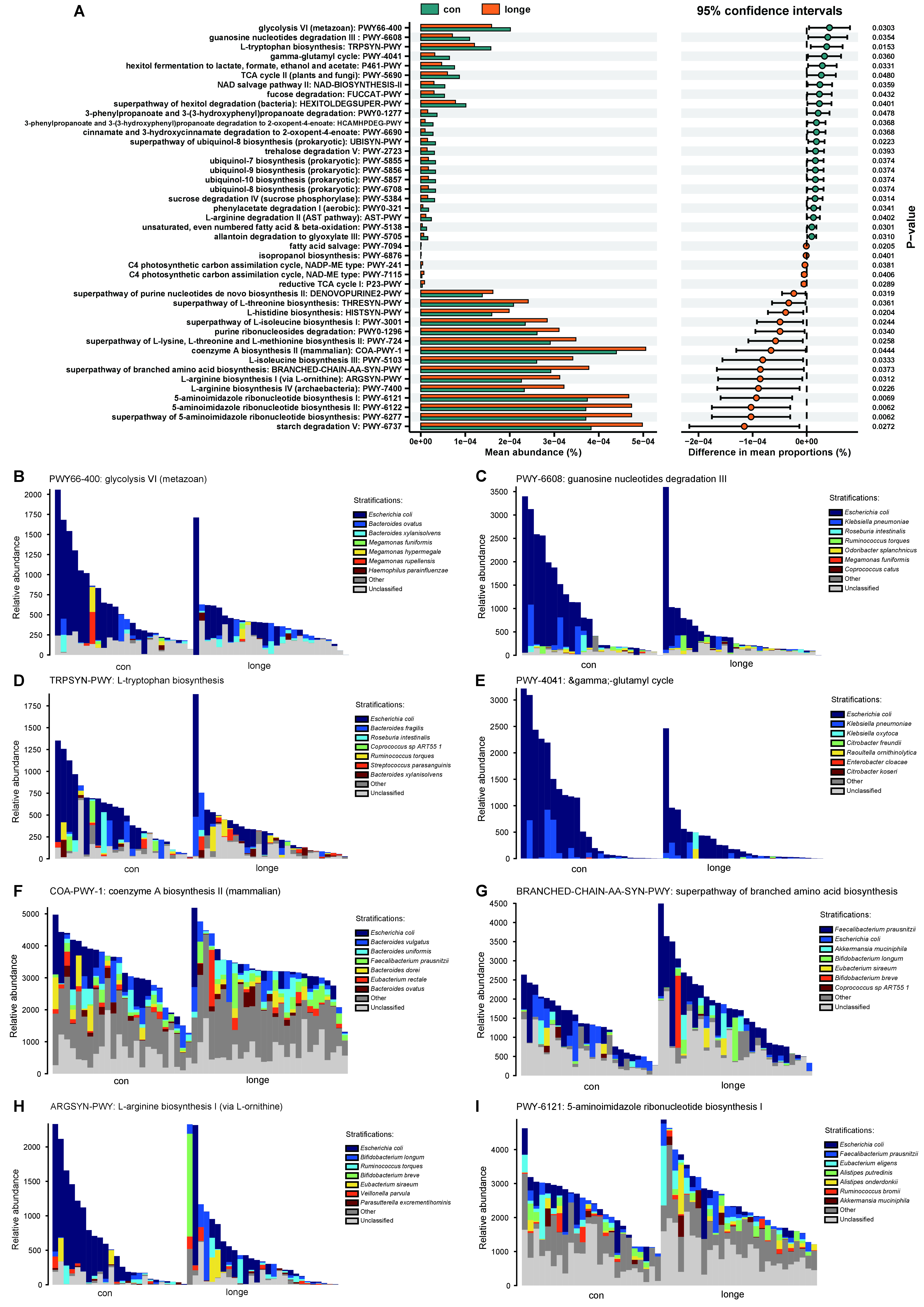


**Figure S3.** Association between clinical factors, frailty, and gut microbiota.


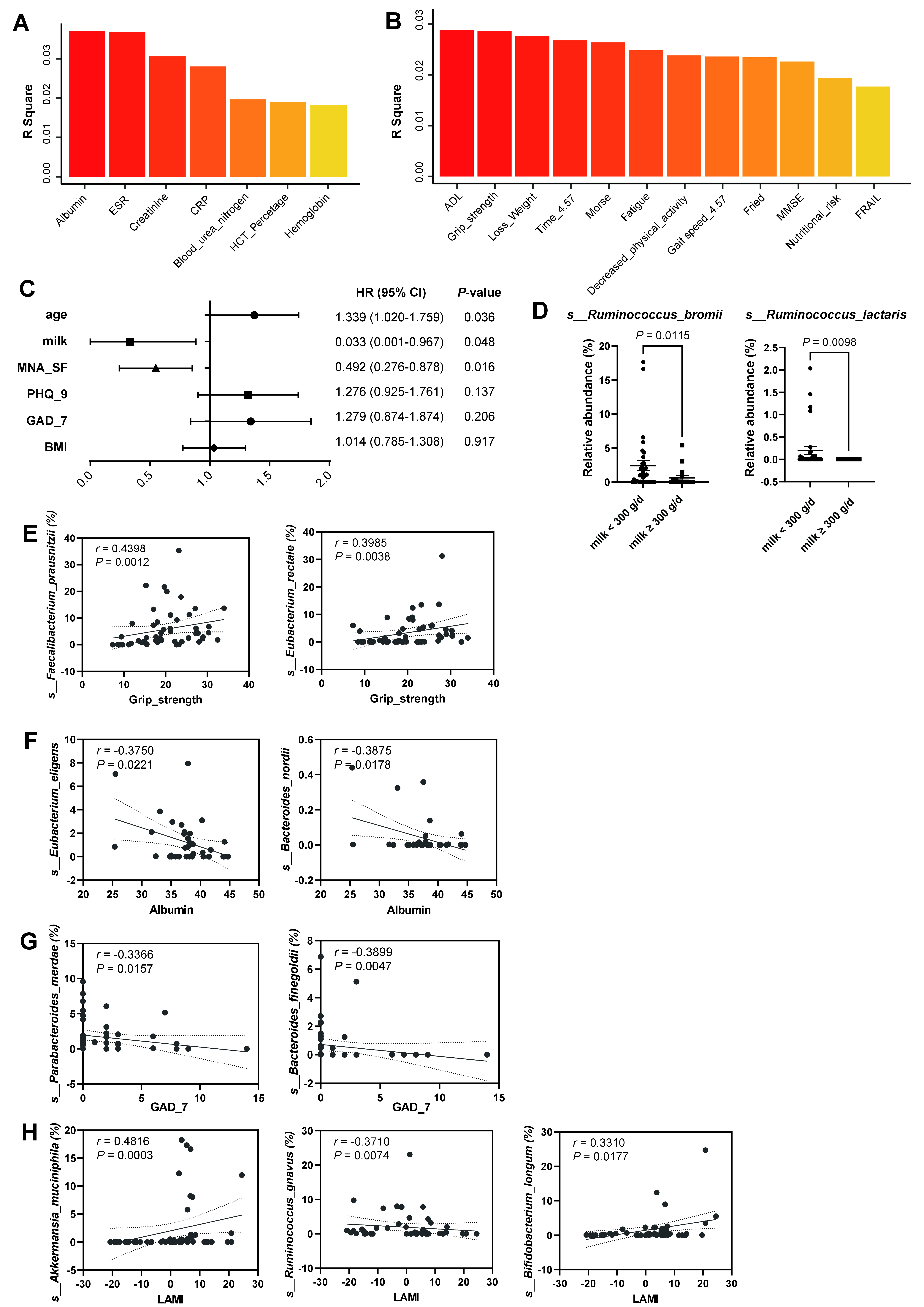


**Figure S4.** Altered pathways in subgroups.


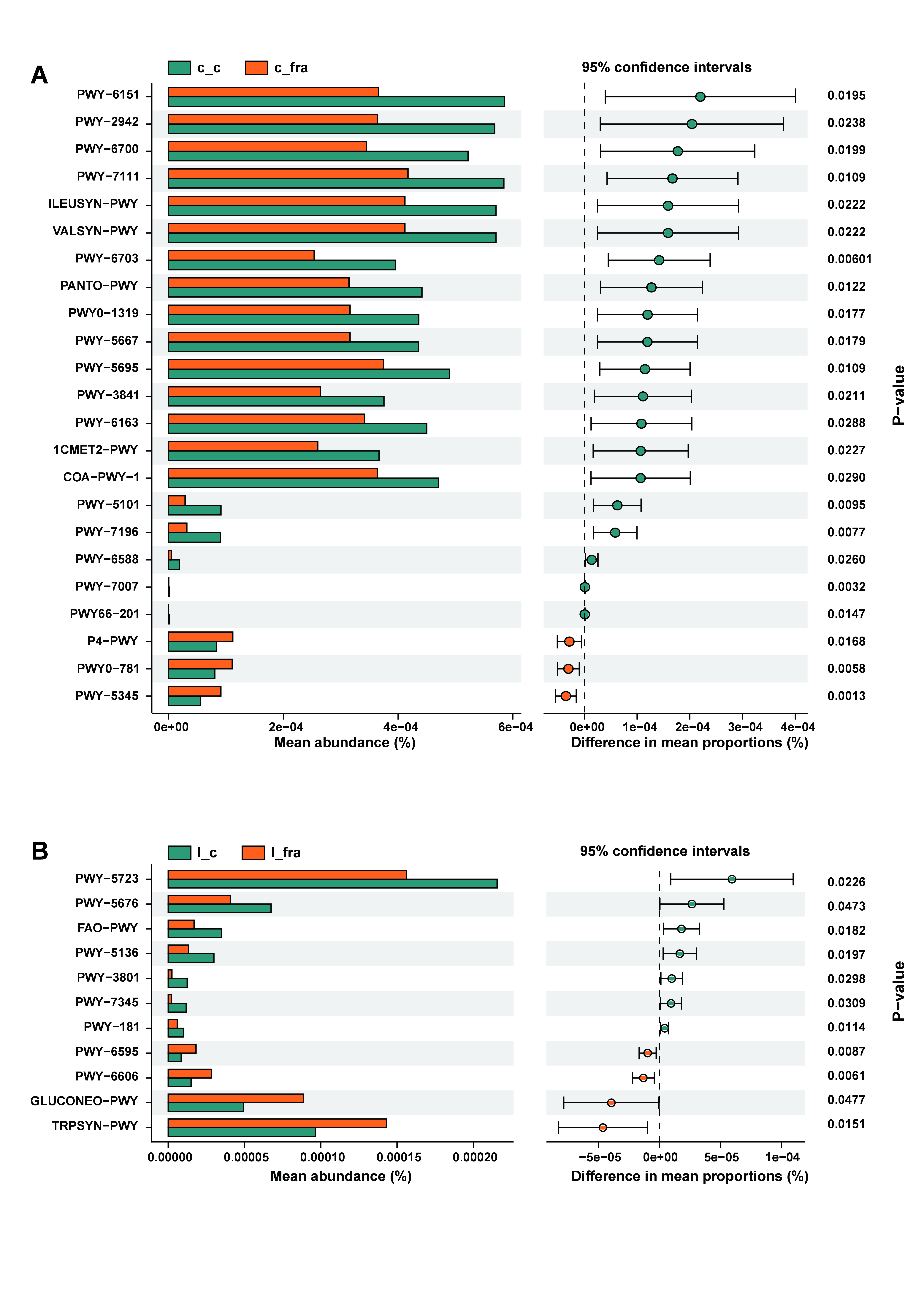


**Figure S5.** Flowchart of study.


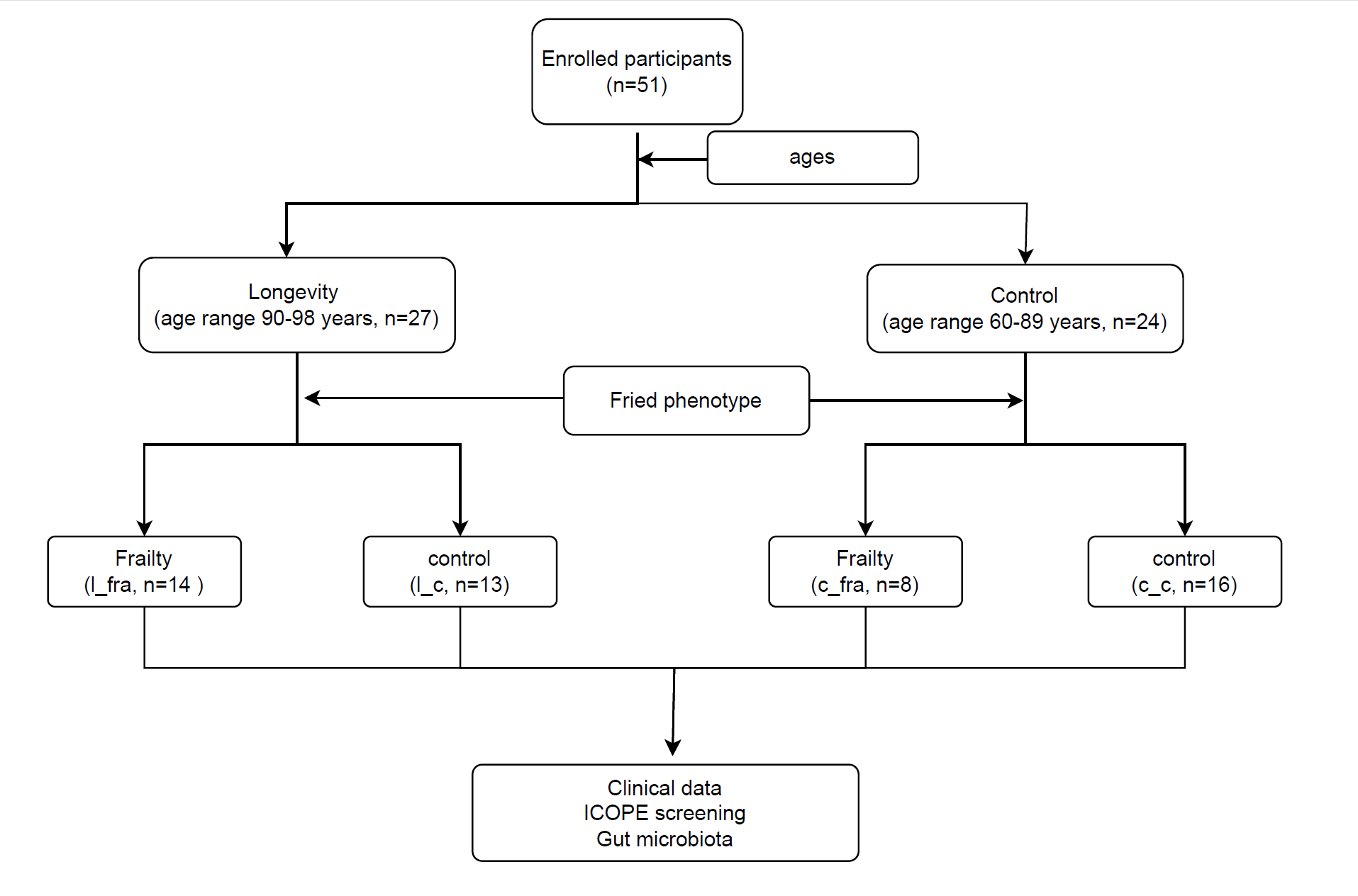

Supplement: Supplementary file 1 [file microorganisms-13-01657-s001.zip › microorganisms-3634894-supplementary figures.docx]
